# Predicting CD137 upregulation on NK cells in patients receiving monoclonal antibody therapy

**DOI:** 10.1186/2051-1426-3-S2-P98

**Published:** 2015-11-04

**Authors:** Amani Makkouk, Vandana Sundaram, Manisha Desai, Cariad Chester, Holden Maecker, Holbrook E Kohrt

**Affiliations:** 1 Stanford University, Stanford, CA, USA

## Background

Monoclonal antibody (mAb) therapy-has changed the natural history of patients with B cell lymphomas, breast cancer, and head and neck cancers. However, response rates are suboptimal, highlighting the need to enhance mAb activity. Combining a tumor targeting mAb with a second antibody that activates natural killer (NK) cells may improve the therapeutic effects of mAbs. Engagement of the Fc receptor (FcR) on NK cells by mAbs coating tumor cells has been previously shown to enhance their expression of CD137 [1-3]. We conducted preliminary analyses as a first step toward developing a nomogram to predict CD137 up-regulation following therapy with mAb.

## Methods

Patient tumor and immune characteristics were collected from patients with Non-Hodgkin's lymphoma, breast cancer, and head and neck cancers receiving mAb therapy as part of clinical trials. CD137 expression pre- and post-mAb therapy was assessed by mass cytometry time of flight (CyTOF) analysis. We examined the difference (post-pre mAB therapy) in CD137 expression for each cancer type. Specifically, we applied multivariate lasso regression tools and used classification and regression trees (CART) to predict post-CD137 expression, separately for each cancer type.

## Results

We analyzed data from 62 patients with breast cancer, 46 with head/neck cancer and 91 with Non-Hodgkin's lymphoma. The difference (post-pre) in CD137 expression values was significantly different for each cancer type (mean (SD): Breast: 6.6 (6.5); Head/Neck: 11.0 (7.0); NHL: 7.5 (7.1), p

## Conclusions

Our preliminary results suggest that FcR polymorphism, pre-treatment expression levels of CD137 and age are significant predictors of CD137 uptake in patients. Further work validating our models will provide the opportunity to develop a nomogram that may be used to individualize this therapeutic approach for patients.
